# Anxiety and depression in newly diagnosed patients with inflammatory bowel disease (the IBSEN III study) compared with the general population in Norway

**DOI:** 10.1093/ecco-jcc/jjag021

**Published:** 2026-03-04

**Authors:** Ingunn Johansen, Milada C Hagen, Stine T Løkkeberg, Tone B Aabrekk, Øyvind Asak, May-Bente Bengtson, Raziye Boyar, Trond Espen Detlie, Svein Oskar Frigstad, Kristina I Aass Holten, Øistein Hovde, Gert Hüppert-Hauss, Charlotte Lund, Asle W Medhus, Bjørn C Olsen, Vibeke Strande, Roald Torp, Simen Vatn, Marte L Høivik, Vendel Kristensen, Lars-Petter Jelsness-Jørgensen, Randi Opheim

**Affiliations:** Institute of Health and Society, Faculty of Medicine, University of Oslo, Oslo, Norway; Department of Health, Welfare and Organization, Østfold University College, Fredrikstad, Norway; Faculty of Health Sciences, Oslo Metropolitan University, Oslo, Norway; Department of Health, Welfare and Organization, Østfold University College, Fredrikstad, Norway; Institute of Clinical Medicine, University of Oslo, Oslo, Norway; Department of Medicine, Vestfold Hospital Trust, Tønsberg, Norway; Department of Gastroenterology, Innlandet Hospital Trust, Lillehammer, Norway; Department of Medicine, Vestfold Hospital Trust, Tønsberg, Norway; Department of Medicine, Diakonhjemmet Hospital, Oslo, Norway; Department of Gastroenterology, Akershus University Hospital, Lørenskog, Norway; Department of Medicine, Vestre Viken Hospital Trust, Bærum, Norway; Institute of Clinical Medicine, University of Oslo, Oslo, Norway; Department of Gastroenterology, Østfold Hospital Trust, Sarpsborg, Norway; Institute of Clinical Medicine, University of Oslo, Oslo, Norway; Department of Medicine, Innlandet Hospital Trust, Gjøvik, Norway; Department of Gastroenterology, Telemark Hospital Trust, Skien, Norway; Institute of Clinical Medicine, University of Oslo, Oslo, Norway; Department of Gastroenterology, Oslo University Hospital, Oslo, Norway; Institute of Clinical Medicine, University of Oslo, Oslo, Norway; Department of Gastroenterology, Oslo University Hospital, Oslo, Norway; Institute of Clinical Medicine, University of Oslo, Oslo, Norway; Department of Gastroenterology, Telemark Hospital Trust, Skien, Norway; Institute of Clinical Medicine, University of Oslo, Oslo, Norway; Unger-Vetlesen Institute, Lovisenberg Diaconal Hospital, Oslo, Norway; Department of Medicine, Innlandet Hospital Trust, Hamar, Norway; Department of Gastroenterology, Akershus University Hospital, Lørenskog, Norway; Department of Medicine, Innlandet Hospital Trust, Gjøvik, Norway; Institute of Clinical Medicine, University of Oslo, Oslo, Norway; Department of Gastroenterology, Oslo University Hospital, Oslo, Norway; Institute of Clinical Medicine, University of Oslo, Oslo, Norway; Department of Gastroenterology, Oslo University Hospital, Oslo, Norway; Department of Health, Welfare and Organization, Østfold University College, Fredrikstad, Norway; Department of Gastroenterology, Østfold Hospital Trust, Sarpsborg, Norway; Institute of Health and Society, Faculty of Medicine, University of Oslo, Oslo, Norway

**Keywords:** IBD, anxiety, depression

## Abstract

**Background and Aims:**

Symptoms of anxiety and depression are common in inflammatory bowel disease (IBD); the aim of this study was to assess the proportion of anxiety and depression in patients newly diagnosed with IBD, compare the rates with the Norwegian general population (NGP), and examine associations with selected sociodemographic, psychological, and disease-related factors.

**Methods:**

This prospective cohort study included newly diagnosed patients with IBD, and data from the HUNT4 survey of the NGP. Anxiety and depression were assessed using the Hospital Anxiety and Depression Scale. Crude statistical comparisons were performed using t-tests, Mann–Whitney U test, chi-square tests, or Fisher’s exact tests. Adjusted associations were modeled using multiple robust linear regression and multiple logistic regression.

**Results:**

In total, 938/1562 (62.1%) patients with IBD completed the Hospital Anxiety and Depression Scale (Crohn’s disease [CD]: *n* = 297, ulcerative colitis [UC]: *n* = 641). The proportion of anxiety was 37.4% in CD and 32.1% in UC, while depression was reported by 21.9% and 16.8%, respectively. Both rates were significantly higher than those observed in the NGP (17.5% for anxiety and 9.4% for depression). Compared with the NGP, males with CD had significantly higher levels of anxiety and depression, males with UC had elevated anxiety only, while females with CD and UC showed increased anxiety and depression. Both substantial fatigue and general self-efficacy were significantly associated with anxiety and depression in IBD.

**Conclusions:**

Newly diagnosed patients with IBD experienced significant psychological challenges compared with the NGP. Early identification of anxiety and depression may enable targeted interventions.

## 1. Introduction

Inflammatory bowel diseases (IBD), comprising Crohn’s disease (CD) and ulcerative colitis (UC), are chronic disorders characterized by an unpredictable disease course with flare-ups and periods of remission.^1^^,^^2^ Common symptoms include bloody stools, diarrhea, pain, and fatigue, all of which can significantly affect health-related quality of life (HRQoL).^3–6^ The chronic nature and severity of IBD, along with its unpredictable course, may adversely affect psychological health and contribute to anxiety and depression.^7^^,^^8^ The European Crohn’s and Colitis Organization guidelines on the diagnosis and management of CD and UC recommend psychological interventions for anxiety and depression.^9^^,^^10^ However, studies indicate that patients with IBD frequently experience unmet psychological needs and perceive limited attention to the psychological aspects of the disease.^11^^,^^12^ In addition, the risk of psychological health concerns is highest during the first year following diagnosis,^13^ when patients are faced with complex medical information, diagnostic procedures, medication adjustments, and therapeutic decision-making.^11^

Anxiety and depression are more prevalent in patients with IBD compared with the general population.^14^ Factors associated with anxiety in IBD include CD diagnosis, self-reported disease activity, disease severity, fatigue, non-adherence to treatment, female gender, unemployment, disability, low socioeconomic status, and lack of family support.^7^^,^^15–17^ Similarly, depression in IBD is associated with self-reported disease activity, age, unemployment, disability, low socioeconomic status, and lack of family support.^7^^,^^15^ Patients with IBD and concomitant anxiety and depression are more likely to use healthcare resources, including hospital- and emergency-room admissions.^8^^,^^18^^,^^19^

To our knowledge, only a few studies have addressed anxiety and depression in patients newly diagnosed with IBD.^20–23^ To the best of our knowledge, no national or international studies have compared anxiety and depression in newly diagnosed IBD with anxiety and depression in the general population. An understanding and awareness of the associations and risk factors related to symptoms of anxiety and depression in patients newly diagnosed with IBD is crucial for effective long-term follow-up.

Thus, this study aimed to determine the proportion of anxiety and depression in patients newly diagnosed with CD and UC, and stratified by gender, to compare these patients with the general Norwegian population (NGP). The secondary aim was to explore the potential associations between anxiety, depression, and selected sociodemographic, psychological, and disease-related factors.

## 2. Methods

### 2.1. Study design and populations

The Inflammatory Bowel South-Eastern Norway III study (IBSEN III) is a prospective population-based inception cohort study that included new cases of IBD and symptomatic non-IBD controls in Southeast Norway, covering approximately 2.95 million inhabitants between 2017 and 2019.^24^ Further details on the IBSEN III study design and patient inclusion have previously been described.^24^ The present study included adult patients (≥18 years) who completed the Hospital Anxiety and Depression Scale (HADS).

### 2.2. Data collection

In the IBSEN III study, standardized clinical, biochemical, endoscopic, and demographic data and patient-reported outcome measures (PROMS) were collected at baseline according to the standard operating procedures of the study. All patients underwent colonoscopy with biopsy at the time of inclusion. Fecal samples for calprotectin analysis were collected from all patients and analyzed in the same laboratory. Fecal calprotectin ≥250 µg/g was defined as active inflammation.^25^ Clinical disease activity was assessed using the Harvey–Bradshaw Index (HBI) for CD^26^ and Simple Clinical Colitis Activity Index (SCCAI) for UC,^27^ where a score of ≥5 on the HBI and ≥3.0 on the SCCAI was defined as active disease for CD and UC, respectively.^28^ For UC, disease extent and severity, and for CD, location and behavior, were categorized based on the Montreal classification.^29^

Sociodemographic data from the IBSEN III study included age, gender, marital status, educational status, smoking status, and work status. Marital status was dichotomized as living together (married/partner) or alone (single, widowed, separated, or divorced). Educational level was dichotomized as higher (>12 years) or primary (≤12 years) education. Work status was dichotomized into work-related (employed/student) and non-work-related (homemaker, disability beneficiary, unemployed, or retired) activities.

### 2.3. NGP—Trøndelag Health Study ([HUNT])

Data from the general population were retrieved from HUNT, a large, population-based cohort study in Norway. Data from the HUNT4 survey of adults ≥20 years were collected between 2017 and 2019.^30^ In addition to the HADS assessing anxiety and depression, sociodemographic data retrieved from the database included age, gender, and education. Educational status was dichotomized as higher (>12 years) or primary (≤12 years) education.

### 2.4. Patient-reported outcome measurements

#### 2.4.1. Hospital Anxiety and Depression Scale

At inclusion, the patients were requested to complete the HADS, a questionnaire developed to assess symptoms of anxiety and depression among diverse clinical and non-clinical hospital populations.^31^ The HADS consists of in total 14 items scored on a four-point Likert scale, and includes two subscales ranging from 0 to 21, one for anxiety (HADS-A) and one for depression (HADS-D). The recall period was 1 week, with higher scores indicating higher symptom burden.^31^ A cut-off score of ≥8 on each subscale was used to indicate the presence of possible anxiety or depressive symptoms.^31^ This threshold is commonly applied in IBD research and in previous HUNT publications, facilitating comparability across studies. The HADS has been translated into Norwegian and validated.^32^ In our study, missing items in the HADS were replaced based on recommended guidelines^33^ with valid scores for both subscales defined as at least five completed items. The sum score for participants who completed five or six items was calculated by multiplying the sum of the completed items by 7/5 or 7/6, respectively.

#### 2.4.2. General Self-Efficacy scale

The General Self-Efficacy scale (GSE) measures optimistic self-belief in coping with general life needs, tasks, and challenges. It consists of 10 statements rated by patients on a four-point scale from 1 “completely agree” to 4 “completely disagree.” The total score is calculated by summing the individual item scores (range: 10-40), with higher scores indicating stronger self-efficacy.^34^ The GSE has demonstrated high reliability and validity^35^^,^^36^ and the Norwegian translation has been validated.^37^^,^^38^

#### 2.4.3. Fatigue questionnaire

The Fatigue Questionnaire (FQ) was developed by Chalder et al.^39^ and measures the extent and severity of fatigue. It consists of 11 items, seven physical and four mental. Each score was dichotomized and summarized, with a score >4 defining substantial fatigue (SF). The questionnaire has been translated into Norwegian and validated^40^ for various groups, including Norwegian patients with IBD.^41^

### 2.5. Statistical analyses

Continuous data are presented as medians and ranges for variables with a skewed distribution and means and standard deviations for normally distributed data. Categorical variables are presented as numbers and percentages. Crude between-group comparisons were performed using independent t-tests for normally distributed data or Mann–Whitney U tests for variables with skewed distributions. Categorical variables were compared using the chi-squared or Fisher’s exact test, as appropriate. The proportion of anxiety and depression was assessed using the HADS, with selected analyses stratified by gender. A cut-off score of ≥8 on each subscale was used to indicate the presence of possible anxiety or depressive symptoms.^31^^,^^42^ We note that HADS is a screening instrument rather than a diagnostic tool; a lower cut-off (≥8) prioritizes sensitivity for case-finding, whereas a higher cut-off (≥11) identifies probable cases with greater specificity. A descriptive table of HADS scores in CD and UC with cut-off of ≥8-10 and ≥11 is provided in the supplementary data. Point estimates are provided with 95% confidence intervals (CIs), derived using binomial approximation.^43^

Robust multiple linear regression analyses were executed to compare HADS scores in patients with CD and UC in IBSEN III with those in the Norwegian general population in HUNT4, and adjusted for age and education. All analyses were performed separately for patients with UC and CD and stratified by gender. Results are reported as regression coefficients with 95% CIs.

Logistic regression analyses were performed to explore the associations between anxiety and depression (dependent variables) and select possible predictive factors. Socio-demographic variables, SF, GSE, and clinical variables with a statistical significance of *P* ≤ .1 in univariate regression analyses were included in the multiple models. Collinearity between potential explanatory variables was assessed. The final multiple model was derived using backward variable selection starting with a full model that included all variables that reached *P* < .10 in univariate analyses. Effect estimates are presented as odds ratios (ORs) with 95% CI. Statistical significance level was set at *P* < .05 for the multiple regression analyses. All analyses were considered exploratory; therefore, no corrections were applied for multiple testing. Data were analyzed using IBM SPSS v.28 (IBM Corp., Armonk, NY, USA) and Stata v.18 (StataCorp., College Station, TX, USA), and Figure 2 was made in R v.4.5.0 (R Core Team, Vienna Austria).

### 2.6. Ethical considerations

This study was approved by the Regional Committee for Medical and Health Research Ethics in Southeast Norway (reference number: 2015/946). The HUNT Research Center at the Norwegian University of Science and Technology obtained permission from the Norwegian Data Inspectorate to store and handle data. Informed consent was obtained from all study participants prior to inclusion in both HUNT and IBSEN III. The IBSEN III study was registered at clinicaltrials.gov (NCT02727959).

## 3. Results

### 3.1. Study populations

**Figure 1.**
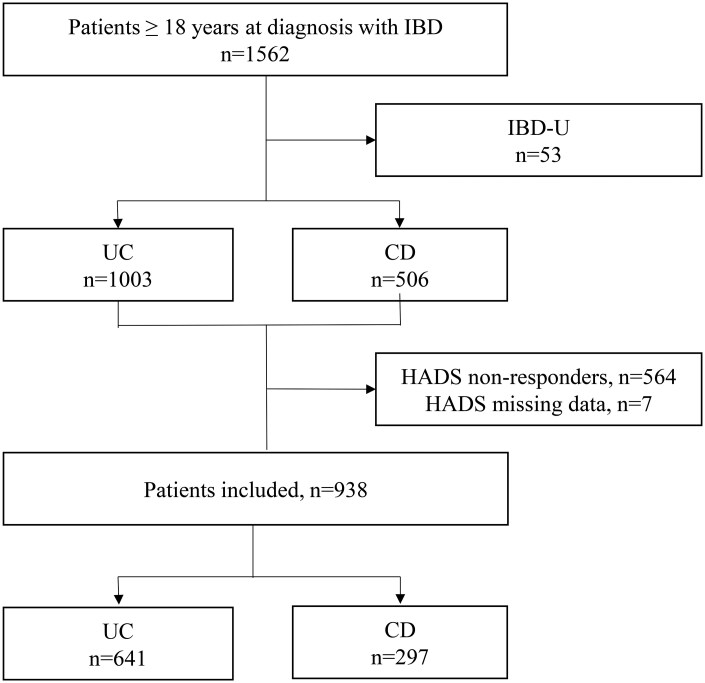
Patient enrolment flowchart. Abbreviations: IBD, inflammatory bowel disease; CD, Crohn’s disease; UC, ulcerative colitis; HADS, Hospital Anxiety and Depression Scale.

In total, 1562 adult (>18 years) patients with IBD were included in IBSEN III (UC: *n* = 1003; CD: *n* = 506) (Figure 1). Of these, 938 (62.1%) completed the HADS at baseline. Compared with non-responders (*n* = 564) or those with incomplete HADS data (*n* = 7), HADS responders included a significantly higher proportion of females (51.3% vs 43.7%, *P* = .004) and a smaller proportion of current smokers (6.8% vs 12.6%, *P* = .004).

Table 1 shows the socio-demographic and clinical characteristics of the IBSEN III and HUNT4 survey cohorts. The distribution of age, gender, and educational level in the patient population differed significantly from that in the general population (*n* = 41 916). Patients with IBD were significantly younger and had a higher level of education, and there was a higher proportion of males among patients with UC.

**Table 1.** Characteristics of the IBSEN III patients and Norwegian general population.

	CD (*n* = 297)	UC (*n* = 641)	NGP (*n* = 41 916)
***Sociodemographic characteristics***			
**Age, mean years (SD)**	39.7 (15.1)	39.6 (14.6)	56.0 (16.9)^b^
**Female sex, *n* (%)**	176 (59.3)	305 (47.6)	23 967 (57.2)^b^
**University ≥2 years, *n* (%)**	138 (46.5)	312 (48.8)	16 604 (39.6)^b^
**Married/co-habitant, *n* (%)**	199 (67.0)	424 (66.6)	
**Working, *n* (%)**	237 (80.1)	532 (83.3)	
**Current smokers, *n* (%)**	31 (10.4)	33 (5.2)	
***Clinical characteristics***			
**HBI >5, *n* (%)**	110 (37.9)		
**SCCAI >2, *n* (%)**		292 (46.7)	
**CD Montreal location**			
** Ileal, *n* (%)**	150 (50.5)		
** Colonic, *n* (%)**	47 (15.8)		
** Ileocolonic, *n* (%)**	100 (33.7)		
** Upper tract only or modifier,** ^a^ *** n* (%)**	7 (2.4)		
**CD Montreal–behavior**			
** Non-stricturing, non-penetrating, *n* (%)**	235 (79.5)		
** Stricturing, *n* (%)**	55 (18.5)		
** Penetrating, *n* (%)**	6 (2.0)		
**Perianal disease,** ^a^ *** n* (%)**	15 (5.1)		
**UC Montreal extent**			
** Proctitis, *n* (%)**		245 (38.3)	
** Left-sided, *n* (%)**		151 (23.6)	
** Extensive, *n* (%)**		243 (38.0)	
**UC Montreal severity**			
** Clinical remission, *n* (%)**		23 (3.6)	
** Mild, *n* (%)**		238 (37.3)	
** Moderate, *n* (%)**		299 (46.9)	
** Severe, *n* (%)**		78 (12.2)	
**Symptom duration in months, median (range min–max) IQR**	9 (0-567) 4-24	4 (0-240) 2-9	
**Calprotectin ≥250 µg/g**	117 (46.2)	249 (46.0)	

Abbreviations: NGP, Norwegian general population; CD, Crohn’s disease; UC, ulcerative colitis; HBI, Harvey–Bradshaw Index; SCCAI, Simple Clinical Colitis Index; IQR, interquartile range.

a Upper tract modifier and perianal disease coexist with other location categories.

b *P*-value estimated between UC and NGP and CD and NGP—all statically significant with *P* < .001.

### 3.2. Proportion of symptoms of anxiety and depression in patients with CD and UC compared with the general population

In the total sample, the proportion of anxiety was 37.4% (95% CI 32.0-43.0%) in patients with CD and 32.1% (95% CI 28.0-35.0%) in patients with UC, whereas depression was reported in 21.9% (95% CI 17.0-27.0) and 16.8% (95% CI 14.0-20.0%) in CD and UC, respectively. No statistically significant differences in anxiety and depression levels were observed between patients with UC and CD, but both diagnostic groups exhibited significantly higher levels of anxiety and depression than those in the NGP (Table 2). A descriptive table of HADS scores in CD and UC with cut-off of ≥8-10 and ≥11 is provided in the supplementary table 1.

**Table 2.** Proportion of anxiety and depression in patients with Crohn’s disease and ulcerative colitis compared with the Norwegian general population.

	Crohn’s disease (*n* = 297), *n* (%) [95% CI]		Ulcerative colitis (*n* = 641), *n* (%) [95% CI]		Norwegian general population (*n* = 41 916), *n* (%) [95% CI]	
**HADS-Anxiety ≥8**	111 (37.4)	[31.9; 43.1]	200 (32.1)	[27.6; 34.9]	7330 (17.5)	[17.1, 17.9]
**HADS-Depression ≥8**	65 (21.9)	[17.3; 27.0]	108 (16.8)	[14.0; 20.0]	3952 (9.4)	[9.2, 9.7]

**Figure 2.**
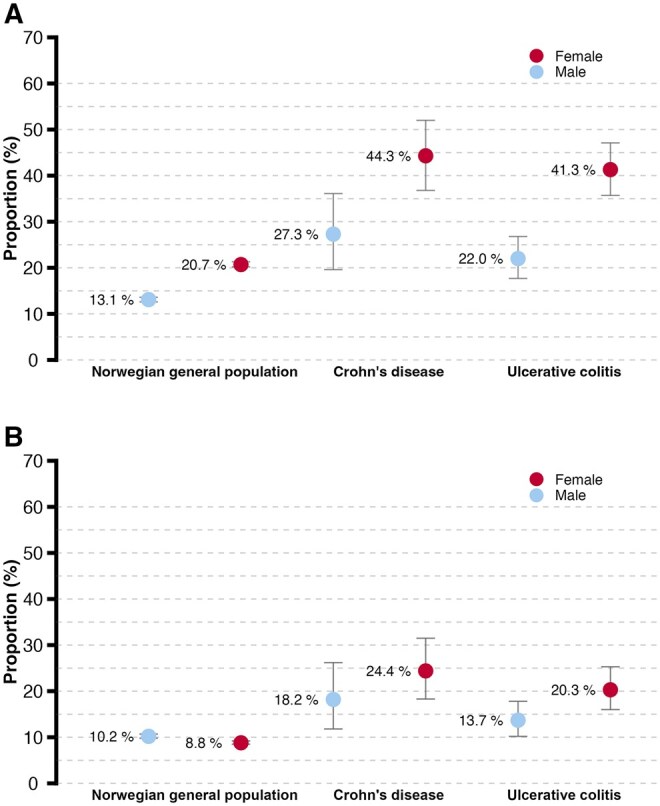
(A) Proportion of anxiety (HADS-A ≥ 8 with 95% CI). Males: Norwegian general population (NGP) 13.1% (12.6-13.6), CD 27.3% (19.6-36.1), UC 22.0% (17.7-26.8). Females: NGP 20.7% (20.2-21.3), CD 44.3% (36.8-52.0), UC 41.3 (35.7-47.1). (B) Proportion of depression (HADS-D ≥ 8 with 95% CI). Males: NGP 10.2% (9.8-10.7), CD 18.2% (11.8-26.2), UC 13.7% (10.2-17.8). Females: NGP 8.8% (8.5-9.2), CD 24.4% (18.3-31.5), UC 20.3% (16.0-25.3)

Figure 2A and B illustrate the proportion of anxiety and depression in the IBD patients compared with the NGP, stratified by gender. Compared with males in the NGP, males with CD had a significantly higher proportion of both anxiety and depression symptoms, whereas males with UC had a significantly higher proportion of symptoms of anxiety, but not depression. Females with CD and UC exhibited a significantly higher proportion of both anxiety and depression symptoms than females in the general population. These gender differences remained statistically significant after adjusting for age and education in the robust multiple linear regression analysis (Table 3).

**Table 3.** Robust multiple regression analyses for HADS-Anxiety and HADS-Depression scores adjusted for age and education.

HADS-Anxiety							HADS-Depression					
	Male			Female			**Male**			Female		
	*B*	95% CI	*P*-value	*B*	95% CI	*P*-value	*B*	95% CI	*P*-value	*B*	95% CI	*P*-value
**Crohn’s disease**	0.8	0.3, 1.4	**.002**	2.2	1.6, 2.7	**<.000**	0.6	1.0, 1.1	**.019**	1.7	1.3, 2.1	**<.000**
**Ulcerative colitis**	0.5	0.2, 0.9	**.002**	1.8	1.4, 2.2	**<.000**	−0.1	−0.4, 0.2	.489	1.1	0.8, 1.4	**<.000**
**NGP (ref)**	0			0			0			0		

Abbreviations: NGP, Norwegian general population; CI, confidence interval.

### 3.3. Factors associated with anxiety and depression in CD

The results of the univariate and multiple logistic regression analyses are presented in Table 4. When adjusted for age, gender, HBI, SF, and GSE, the final multiple logistic regression models indicated that SF increased the odds of anxiety fourfold (OR = 4.1 [95% CI 2.1-7.8]), whereas GSE scores reduced the odds by 10% (OR = 0.9 [95% CI 0.9-0.9]). When adjusted for age, marital status, education, work status, HBI, SF, and GSE, the final multiple logistic regression model indicated that patients with SF had a more than 12 times higher odds of depression (OR = 12.3 [95% CI 3.5-43.3]). Moreover, unemployed patients had a nearly four times higher odds for depression (OR = 3.8 [95% CI: 1.7-8.,6]) compared to employed patients, whereas GSE scores reduced the odds by 10% (OR = 0.9 [95% CI 0.8-1.0]).

**Table 4.** Variables associated with anxiety and depression (HADS score >8) in patients with Crohn’s disease (*n* = 297). Results from multiple logistic regression analyses with stepwise backward selection.

		**HADS-Anxiety >8**							**HADS-Depression >8**					
		Univariate			Multiple				Univariate			Multiple		
Variable	*n*	OR	95% CI	*P*-value	OR	95% CI	*P*-value	*n*	OR	95% CI	*P*-value	OR	95% CI	*P*-value
**Age**	111	0.98	0.97-1.00	.020				65	0.98	0.96-1.00	.016			
**Sex**														
** Males (ref)**	33	1						22	1					
** Females**	78	2.12	1.29-3.49	.003				43	1.45	0.82-2.59	.200			
**Marital status**														
** Living together (ref)**	70	1						37	1					
** Living alone**	41	1.33	0.81-2.18	.265				38	1.75	1.00-3.09	.052			
**Education**														
** >12 years (ref)**	50	1						21	1					
** <12 years**	61	1.10	0.68-1.76	.705				44	2.13	1.19-3.81	.011			
**Work status**														
** Working (ref)**	85	1						42	1					
** Not working**	26	1.41	0.79-2.51	.245				23	2.97	1.60-5.52	<.001	3.77	1.65-8.63	.002
**HBI**														
** <5 (ref)**	59	1						31	1					
** ≥5**	50	1.71	1.05-2.78	.031				33	2.06	1.17-3.61	.012			
** CD behavior**														
** B1 non-stricturing Non-penetrating (ref)**	92	1						55	1					
** B2 stricturing**	18	0.76	0.41-0.42	.390				9	0.64	0.30-1.40	.266			
** B3 penetrating**	1	0.31	0.04-2.72	.293				1	0.66	0.08-5.75	.705			
** B4 behavior**	5	0.83	0.28-2.50	.740				3	0.89	0.24-3.24	.856			
**CD location**														
** L1 ileum (ref)**	56	1						34	1					
** L2 colon**	18	1.04	0.53-2.05	.905				9	0.81	0.36-1.84	.611			
** L3 ileo-colon**	37	0.99	0.58-1.66	.960				22	0.96	0.52-1.77	.901			
L4 upper*	2	0.66	0.13-3.48	.628	O			3	2.76	0.60-12.65	.192			
**Symptom duration in months**	111	1.00	1.00-1.01	.218				64	1.00	1.00-1.01	.653			
**Calprotectin**														
** <250 µg/g (ref)**	48	1						29	1					
** ≥250 µg/g**	48	1.28	0.77-2.12	.349				26	1.05	0.58-1.92	.863			
**Substantial fatigue**														
** FQ score <4 (ref)**	15	1						3	1					
** FQ score >4**	93	4.35	2.34-8.11	<.001	4.08	2.14-7.77	<.001	60	12.41	3.77-40.82	<.001	12.30	3.50-43.26	<.001
**GSE**	111	0.90	0.86-0.94	<.001	0.91	0.86-0.95	<.001	65	0.87	0.83-0.92	<.001	0.89	0.84-0.95	<.001

Abbreviations: HADS, Hospital Anxiety and Depression Scale; CD, Crohn’s disease; HBI, Harvey–Bradshaw Index; GSE, The General Self-Efficacy Scale; OR, odds ratio; CI, confidence interval; FQ, Fatigue Questionnaire. *L4 is a modifier that can be added to L1‒L3 when upper gastrointestinal disease is present. Multiple regression: the final multiple model was derived using backward variable selection starting with a full model that included all variables that reached *P* < .10 in univariate analyses. Effect estimates are presented as OR with 95% CI.

### 3.4. Factors associated with anxiety and depression in UC

The results of the univariate and multiple logistic regression analyses for patients with UC are presented in Table 5. When adjusted for age, gender, disease extent, SF, and GSE scores, the final multiple logistic regression models showed that female gender increased the odds of anxiety by 50% (OR = 1.5 [95% CI 1.0-2.3]), and SF increased the odds of anxiety fourfold (OR: 4.5 [95% CI 2.8-7.1]). Disease extent (left-sided colitis) and GSE scores were associated with reduced odds of anxiety. When adjusted for gender, education, work status, SF, and GSE scores, patients with SF had a more than 10 times higher odds of depression (OR = 10.2 [95% CI 4.6-22.7]), whereas higher GSE scores significantly reduced the risk of depression by 10% (OR = 0.9 [95% CI 0.8-0.9]).

**Table 5.** Variables associated with anxiety and depression (HADS score >8) in patients with ulcerative colitis (*n* = 641). Results from multiple logistic regression analyses with stepwise backward selection (*n* = 641).

		**HADS-Anxiety >8**							HADS-Depression >8					
		Univariate			Multiple				Univariate			Multiple		
Variable	*n*	OR	95% CI	*P*-value	OR	95% CI	*P*-value	*n*	OR	95% CI	*P*-value	OR	95% CI	*P*-value
**Age**	200	0.98	0.97-0.99	.003	0.98	0.97-1.00	.019	108	1.0	0.98-1.01	.842			
**Sex**														
** Males (ref)**	74	1						46	1					
** Females**	126	2.49	1.77-3.52	<.001	1.52	1.01-2.28	.042	62	1.61	1.06-2.44	.026			
**Marital status**														
** Living together (ref)**	124	1						67	1					
** Living alone**	74	1.29	0.91-1.83	.158				41	1.27	0.83-1.95	.275			
**Education**														
** >12 years (ref)**	96	1						44	1					
** <12 years**	103	1.03	0.74-1.44	.860				64	1.48	0.97-2.25	.069			
**Work status**														
** Working (ref)**	160	1						80	1					
** Not working**	39	1.33	0.86-2.06	.195				28	2.00	1.22-3.28	.006			
**SSCAI**														
** <3 (ref)**	94	1						50	1					
** ≥3**	98	1.28	0.91-1.81	.150				54	1.28	0.84-1.96	.245			
**Severity**														
** Moderate (ref)**	53	1						100	1					
** Mild**	38	0.85	0.59-1.12	.128				71	0.88	0.56-1.39	.589			
** Remission**	2	0.42	0.14-1.26	.372				4	0.44	0.10-1.94	.280			
** Severe**	15	0.94	0.55-1.60	.816				25	1.11	0.59-2.09	.758			
**Disease extent**														
** E1 (ref)**	88	1						42	1					
** E2**	39	0.62	0.40-0.97	.037	0.56	0.33-0.96	.035	25	0.96	0.56-1.65	.880			
** E3**	73	0.77	0.77-1.12	.168				41	0.98	0.61-1.57	.937			
**Symptom duration in months**	200	1.00	0.99-1.01	.368				108	1.00	0.992-1.01	.800			
**Calprotectin**														
** <250 µg/g (ref)**	96	1						47	1					
** ≥250 µg/g**	69	0.77	0.53-1.11	.160				37	0.91	0.57-1.45	.692			
**Substantial fatigue**														
** FQ score <4 (ref)**	5	1						4	1					
** FQ score >4**	195	5.39	3.15-8.28	<.001	4.47	2.81-7.12	< .001	104	11.90	5.42-26.13	< .001	10.17	4.55-22.74	<.001
**GSE**	200	0.86	0.84-0.89	<.001	0.87	0.84-0.90	< .001	108	0.89	0.89-0.84	< .001	0.87	0.83-0.90	<.001

Abbreviations: HADS, Hospital Anxiety and Depression Scale; UC, ulcerative colitis; GSE, The General Self-Efficacy Scale; OR, odds ratio; CI, confidence interval; SSCAI, Simple Clinical Colitis Activity Index. Multiple regression: only variables from the univariate analyses with *P*-value ≤.1. Stepwise backward selection was performed to evaluate the strength of the association between the independent and dependent variables. Effect estimates are presented as OR with 95% CI.

## 4. Discussion

In this study among newly diagnosed patients with IBD, we observed a significantly higher proportion of symptoms of anxiety and depression compared with the NGP. These findings are consistent with a systematic review on elevated rates of anxiety and depression in patients with IBD relative to the general population.^14^ In general, The HUNT population is considered to be representative of the NGP,^44^ but the distribution of age, gender, and educational level in the present patient population differed significantly from that in the general population in HUNT4. Our analyses of anxiety and depression based on gender and diagnosis, as well as comparisons with the NGP, and the use of robust regression analyses adjusted for age and educational level, strengthen the validity of our findings. To our knowledge, studies comparing newly diagnosed patients with IBD and general population are scarce. Our study revealed gender-related differences. Compared to the NGP, newly diagnosed females with IBD had significantly higher proportion of both anxiety and depression, whereas males with IBD had a significantly higher proportion of anxiety symptoms, but not depression. Our findings may reflect the challenging situation of being diagnosed with a chronic disease for both genders, and further research is needed to investigate whether these findings persist or change over time. Moreover, a bidirectional relationship between IBD, anxiety, and depression has been suggested, with evidence indicating an increased risk of depression and anxiety both before and after IBD diagnosis.^45^ No significant differences in the proportion of symptoms of anxiety and depression were observed between patients with CD and UC, but when analyzed by gender, women with IBD had a significantly higher proportion of anxiety symptoms, aligning with earlier reviews.^7^^,^^14^^,^^46^

The proportions of anxiety and depression align with a prospective multi-center cohort study of patients newly diagnosed with IBD (<6 months), which also employed the HADS with a cut-off score of ≥8 to indicate symptoms of anxiety or depression.^20^ In addition, a small study of newly diagnosed patients with IBD reported lower rates than those observed in our study,^21^ while another study focusing on patients with UC found a higher proportion of depression than that observed in this study.^47^ A recent study^23^ applied a more rigorous HADS cut-off score of ≥11 when assessing symptoms of anxiety and depression in newly diagnosed patients. We chose a HADS cut-off of ≥8 to identify possible symptoms of anxiety and depression, consistent with prior IBD and population studies. This approach favors case-finding^42^ and comparability but will yield higher proportion estimates than a more conservative HADS cut-off (≥11), which is often used to indicate probable clinical cases. Using the more rigorous cut-off yielded lower proportions of anxiety and depression symptoms in our study compared to those reported in the comparative study.^23^

However, comparing the findings from these studies with selected samples to those of our population-based cohort is complicated by differences in sample size, disease severity, and HADS cut-off scores, and neither of these studies compared rates with general populations.

In our study, substantial fatigue and self-efficacy were the strongest predictive factors independently associated with symptoms of anxiety and depression. Fatigue is a common symptom of many chronic illnesses, including IBD, with nearly two-thirds of patients newly diagnosed with IBD reporting substantial fatigue.^48^ Fatigue is considered to be one of the most troublesome symptoms experienced by patients with IBD^49^^,^^50^ and negatively affects HRQoL,^51^ thereby also affecting the patients’ psychological well-being. Fatigue is also associated with anxiety and depression in IBD.^48^^,^^52–54^ The pathogenesis of fatigue remains unclear. However, psychological morbidities have been suggested as contributing factors,^55^ and there is a considerable symptomatic overlap between fatigue, anxiety, and depression.^16^^,^^54^^,^^56^ Nevertheless, our findings highlight the importance of recognizing fatigue as a potential contributor to anxiety and depression symptoms in newly diagnosed patients, despite limited treatment options owing to its unclear and multifactorial pathogenesis.^55^ In our study, patients with CD and substantial fatigue had a 12 times higher odds for symptoms of depression, but the wide CIs (95% CI: 3.5-43.3) indicate considerable uncertainties, probably related to the low number of patients with CD and depression.

General self-efficacy refers to an individual’s belief in their ability to handle challenges^57^ and is associated with vulnerability to psychological distress.^57^^,^^58^ In addition, self-efficacy has been associated with effective self-management and self-esteem^59^^,^^60^ and studies in other chronic conditions have demonstrated that higher self-efficacy scores are associated with improved health outcomes.^61–63^ Our results suggest that higher self-efficacy is associated with lower odds of anxiety and depression symptoms, consistent with findings from other studies in patients with IBD.^64^^,^^65^ According to Bandura, self-efficacy is a modifiable factor,^57^ suggesting that interventions aimed at increasing self-efficacy may be particularly relevant for patients newly diagnosed with chronic diseases, such as IBD.

Our multiple logistic regression analyses indicated that work status was significantly associated with symptoms of depression in patients with CD. An inability to participate in work life may affect psychological well-being, and this finding partially aligns with previous studies,^15^^,^^66^ and a recent systematic review and meta-analysis^67^ on work impairment found that patients with CD were more negatively affected compared with patients with UC.

Interestingly, even though approximately 50% had active disease at diagnosis in our study, we did not find an association between IBD disease activity and the proportion of depression and anxiety symptoms, in contrast to previous systematic reviews.^7^^,^^14^ Contrary to the newly diagnosed patients in our study, these reviews included patients with a wide range of disease durations, which makes direct comparison challenging. This finding emphasizes the need to focus on the patient’s symptoms, in addition to disease activity, in the follow-up of newly diagnosed patients. Early detection of psychological distress, regardless of disease activity, provides an opportunity for personalized symptom management and support. This is in accordance with recommendations in Mikocka-Walus et al. with an integrated biopsychosocial care model including regular mental health screenings.^68^

A major strength of this study is its large, population-based sample and the comparison of newly diagnosed patients with IBD with age- and gender-adjusted cohorts from the known NGP of HUNT4, providing a novel and unique insight into this patient population. Contrary to our study, disease activity in IBD studies is often assessed using clinical disease activity indices derived from self-reported data rather than biomarkers.^7^ Therefore, the inclusion of fecal calprotectin as a biomarker in this study is a notable strength. This study also has its limitations. Recall bias associated with PROMS cannot be entirely excluded. However, the large number of patients included in the IBSEN III study, and the similarity between HADS responders and non-responders across most background variables, strengthen our findings and support the representativeness of the study population with IBD. Furthermore, no data on symptoms of anxiety and depression among the IBSEN III patients prior to inclusion were available. Finally, the limited sample size of patients with CD and depression may limit the generalization of these results to the general CD patient population. It is also important to emphasize that the HADS is a screening tool, not a diagnostic tool, and HADS scores ≥8 do not indicate that the criteria for a clinical diagnosis of anxiety or depression (eg, ICD-10 or DSM-5) are met.

In conclusion, this study provides valuable insights into the psychological challenges faced by patients newly diagnosed with IBD, emphasizing the importance of comprehensive care that addresses both the physical and psychological aspects of this chronic disease. Recognizing the gender-specific aspects of IBD is important for improving disease management and promoting personalized care. Such interventions may include addressing substantial fatigue, enhancing self-efficacy, and providing psychosocial support. Future longitudinal studies should explore the relationships between disease activity, psychological treatment regimen and interventions, as well as psychological outcomes to further advance understanding and improve treatment strategies. The findings in this study emphasize the importance of integrating psychological health screening and support into the routine care of newly diagnosed patients with IBD. Healthcare providers should prioritize early identification of psychological health challenges to enable timely and targeted interventions.

## Supplementary Material

jjag021_Supplementary_Data

## Data Availability

The data for this study are stored on a secure server for sensitive data managed by the University of Oslo, Norway. The data underlying this article cannot be shared publicly due to the privacy of individuals who participated in the study. The data will be shared on reasonable request to the corresponding author.
