# Supplementary material for: Anxiety and depression in newly diagnosed patients with inflammatory bowel disease (the IBSEN III study) compared with the general population in Norway
Source: J Crohns Colitis. 2026 Mar 4;20(3):jjag021. doi: 10.1093/ecco-jcc/jjag021 (PMC13016783; doi:10.1093/ecco-jcc/jjag021)
Supplement: jjag021_Supplementary_Data [file jjag021_supplementary_data.zip › XXXMain_manuscript_clean_0212225.docx]

**Anxiety and depression in newly diagnosed patients with inflammatory bowel disease (the IBSEN III study) compared with the general population in Norway.**

**Authors:** Ingunn Johansen^1,2^, Milada C. Hagen^3^, Stine T. Løkkeberg^2^, Tone B. Aabrekk^4,5^ Øyvind Asak^6^, May-Bente Bengtson^5^, Raziye Boyar^7^, Trond Espen Detlie^8^, Svein Oskar Frigstad^9^, Kristina I. Aass Holten^4,10^, Øistein Hovde^4,11^, Gert Hüppert-Hauss^12^, Charlotte Lund^4,13^, Asle W. Medhus^4,13^, Bjørn C. Olsen^4,12^, Vibeke Strande^4,14^, Roald Torp^15^, Simen Vatn^8,11^, Marte L. Høivik^4,13^, Vendel Kristensen^4,13^, Lars-Petter Jelsness-Jørgensen^10,16^, Randi Opheim^1^

^1^ Institute of Health and Society, Faculty of Medicine, University of Oslo, Oslo, Norway

^2^ Department of Health, Welfare and Organization, Østfold University College, Fredrikstad, Norway

^3^Faculty of Health Sciences, Oslo Metropolitan University, Oslo, Norway

^4^Institute of Clinical Medicine, University of Oslo, Oslo, Norway

^5^Department of Medicine, Vestfold Hospital Trust, Tønsberg, Norway

^6^Department of Gastroenterology, Innlandet Hospital Trust, Lillehammer, Norway

^7^Department of Medicine, Diakonhjemmet Hospital, Oslo, Norway

^8^Department of Gastroenterology, Akershus University Hospital, Lørenskog, Norway

^9^Department of Medicine, Vestre Viken Hospital Trust, Bærum, Norway

^10^Department of Gastroenterology, Østfold Hospital Trust, Sarpsborg, Norway

^11^Department of Medicine, Innlandet Hospital Trust, Gjøvik, Norway

^12^Department of Gastroenterology, Telemark Hospital Trust, Skien, Norway

^13^Department of Gastroenterology, Oslo University Hospital, Oslo, Norway

^14^Unger-Vetlesen Institute, Lovisenberg Diaconal Hospital, Oslo, Norway

^15^Department of Medicine, Innlandet Hospital Trust, Hamar, Norway

^16^Østfold University College, Halden, Norway

**Short title:** Anxiety and depression in newly diagnosed IBD patients

**List of abbreviations:** IBD, Inflammatory bowel disease; CD, Crohn’s disease; UC, ulcerative colitis; HUNT, Trøndelag Health Study; HADS, Hospital Anxiety and Depression Scale; SF, substantial fatigue; GSE, General Self-Efficacy Scale.

**Correspondence:**

Ingunn Johansen

Høgskolen i Østfold

Postboks 700, N-1757 Halden

Norway

Telephone number: +4769608755

Email: [ingunn.johansen@hiof.no](mailto:ingunn.johansen@hiof.no)

**Author`s contact e-mails:**

Milada C. Hagen: [milasm@oslomet.no](mailto:milasm@oslomet.no)

Stine T. Løkkeberg: stine.t.lokkeberg@hiof.no

Øyvind Asak: oasak@hotmail.com

May-Bente Bengtson: maybente.bengtson@gmail.com

Tone B. Aabrekk: tonebergene.aabrekk@siv.no

Raziye Boyar: [raziye.boyar@diakonsyk.no](mailto:raziye.boyar@diakonsyk.no)

Trond Espen Detlie: trond.espen.detlie@gmail.com

Svein Oskar Frigstad: [svosfr@vestreviken.no](mailto:svosfr@vestreviken.no)

Kristina A. Holten: [kristina.holten@gmail.com](mailto:kristina.holten@gmail.com)

Øistein Hovde: [oistein.hovde@sykehuset-innlandet.no](mailto:oistein.hovde@sykehuset-innlandet.no)

Gert Hüppert-Hauss: [hhge@sthf.no](mailto:hhge@sthf.no)

Charlotte Lund: [charllu@uio.no](mailto:charllu@uio.no)

Asle W. Medhus: [a.w.medhus@medisin.uio.no](mailto:a.w.medhus@medisin.uio.no)

Bjørn C. Olsen: [olsenb@sthf.no](mailto:olsenb@sthf.no)

Vibeke Strande: [vibeke.strande@lds.no](mailto:vibeke.strande@lds.no)

Roald Torp: [roald.a.torp@sykehuset-innlandet.no](mailto:roald.a.torp@sykehuset-innlandet.no)

Simen Vatn: [bikkjas@hotmail.com](mailto:bikkjas@hotmail.com)

Vendel Kristensen: venkri@ous-hf.no

Marte L. Høivik: m.l.hoivik@medisin.uio.no

Lars-Petter Jelsness-Jørgensen: lars.p.jelsness-jorgensen@hiof.no

Randi Opheim: randi.opheim@medisin.uio.no

**ABSTRACT**

Background and Aims:

Symptoms of anxiety and depression are common in inflammatory bowel disease (IBD); the aim of this study was to assess the proportion of anxiety and depression in patients newly diagnosed with IBD, compare the rates with the Norwegian general population, and examine associations with selected sociodemographic, psychological, and disease-related factors.

Methods:

This prospective cohort study included newly diagnosed patients with IBD, and data from the HUNT4 survey of the Norwegian general population (NGP). Anxiety and depression were assessed using the Hospital Anxiety and Depression Scale. Crude statistical comparisons were performed using t-tests, Mann‒Whitney U test, chi-square tests, or Fisher’s exact tests. Adjusted associations were modelled using multiple robust linear regression and multiple logistic regression.

Results:

In total, 938/1562 (62.1%) patients with IBD completed the Hospital Anxiety and Depression Scale (CD: n=297, UC: n=641). The proportion of anxiety was 37.4% in CD and 32.1% in UC, while depression was reported by 21.9% and 16.8%, respectively. Both rates were significantly higher than those observed in the NGP (17.5% for anxiety and 9.4% for depression). Compared with the NGP, males with CD had significantly higher levels of anxiety and depression, males with UC had elevated anxiety only, while females with CD and UC showed increased anxiety and depression. Substantial fatigue and general self-efficacy were both significantly associated with anxiety and depression in IBD.

Conclusions:

Newly diagnosed with IBD experienced significant psychological challenges compared with the NGP. Early identification of anxiety and depression may enable targeted interventions.

Keywords: IBD, anxiety, depression

**Introduction**

Inflammatory bowel diseases (IBD), comprising Crohn’s disease (CD) and ulcerative colitis (UC), are chronic disorders characterized by an unpredictable disease course with flare-ups and periods of remission.^1,2^ Common symptoms include bloody stools, diarrhea, pain, and fatigue, all of which can significantly affect health-related quality of life (HRQoL).^3-6^ The chronic nature and severity of IBD, along with its unpredictable course, may adversely affect psychological health and contribute to anxiety and depression.^7,8^ The European Crohn’s and Colitis Organization guidelines on the diagnosis and management of CD and UC recommend psychological interventions for anxiety and depression.^9,10^ However, studies indicate that patients with IBD frequently experience unmet psychological needs and perceive limited attention to the psychological aspects of the disease.^11,12^ In addition, the risk of psychological health concerns is highest during the first year following diagnosis,^13^ when patients are faced with complex medical information, diagnostic procedures, medication adjustments, and therapeutic decision-making.^11^

Anxiety and depression are more prevalent in patients with IBD compared with the general population.^14^ Factors associated with anxiety in IBD include CD diagnosis, self-reported disease activity, disease severity, fatigue, non-adherence to treatment, female gender, unemployment, disability, low socioeconomic status, and lack of family support.^7,15-17^ Similarly, depression in IBD is associated with self-reported disease activity, age, unemployment, disability, low socioeconomic status, and lack of family support.^7,15^ Patients with IBD and concomitant anxiety and depression are more likely to use healthcare resources, including hospital- and emergency-room admissions.^8,18,19^

To our knowledge, only a few studies have addressed anxiety and depression in patients newly diagnosed with IBD.^20-23^ To the best of our knowledge, no national or international studies have compared anxiety and depression in newly diagnosed IBD with anxiety and depression in the general population. Understanding and awareness of the associations and risk factors related to symptoms of anxiety and depression in patients newly diagnosed with IBD is crucial for effective long -term follow-up.

Thus, this study aimed to determine the proportion of anxiety and depression in patients newly diagnosed with CD and UC, and stratified by gender, compare these patients with the general Norwegian population. The secondary aim was to explore the potential associations between anxiety, depression, and selected sociodemographic, psychological, and disease-related factors.

**METHOD**

**Study design and populations**

The Inflammatory Bowel South-Eastern Norway III study (IBSEN III) is a prospective population-based inception cohort study that included new cases of IBD and symptomatic non-IBD controls in South Eastern Norway, covering approximately 2.95 million inhabitants between 2017 and 2019.^24^ Further details on the IBSEN III study design and patient inclusion have previously been described.^24^ The present study included adult patients (≥ 18 years) who completed the Hospital Anxiety and Depression Scale (HADS).

**Data collection**

In the IBSEN III study, standardized clinical, biochemical, endoscopic, and demographic data and patient-reported outcome measures (PROMS) were collected at baseline according to the standard operating procedures of the study. All patients underwent colonoscopy with biopsy at the time of inclusion. Fecal samples for calprotectin analysis were collected from all patients and analyzed in the same laboratory. Fecal calprotectin ≥ 250 µg/g was defined as active inflammation ^25^. Clinical disease activity was assessed using the Harvey-Bradshaw Index (HBI) for CD ^26^ and simple clinical colitis activity index (SCCAI) for UC,^27^ where a score of ≥ 5 on the HBI and ≥ 3.0 on the SCCAI was defined as active disease for CD and UC, respectively.^28^ For UC, disease extent and severity, and for CD, location and behavior, were categorized based on the Montreal classification.^29^

Sociodemographic data from the IBSEN III study included age, gender, marital status, educational status, smoking status, and work status. Marital status was dichotomized as living together (married/partner) or alone (single, widowed, separated, or divorced). Educational level was dichotomized as higher (> 12 years) or primary (< 12 years) education. Work status was dichotomized into work-related (employed/student) and non-work-related (homemaker, disability beneficiary, unemployed, or retired) activities.

**Norwegian general population (Trøndelag Health Study ([HUNT])**

Data from the general population were retrieved from HUNT, a large, population-based cohort study in Norway. Data from the HUNT4 survey of adults ≥ 20 years were collected between 2017 and 2019.^30^ In addition to the Hospital Anxiety and Depression Scale (HADS) assessing anxiety and depression, sociodemographic data retrieved from the database included age, gender, and education. Educational status was dichotomized as higher (> 12 years) or primary (< 12 years) education.

**Patient-reported outcome measurements**

***Hospital Anxiety and Depression Scale (HADS)***

At inclusion, the patients were requested to complete the HADS, a questionnaire developed to assess symptoms of anxiety and depression among diverse clinical and nonclinical hospital populations.^31^ The HADS consists of in total 14 items scored on a 4-point Likert scale, and includes two subscales ranging from 0 to 21, one for anxiety (HADS-A) and one for depression (HADS-D). The recall period was 1 week, with higher scores indicating higher symptom burden.^31^ A cut‑off score of ≥8 on each subscale was used to indicate the presence of possible anxiety or depressive symptoms.^31^ This threshold is commonly applied in IBD research and in previous HUNT publications, facilitating comparability across studies. The HADS has been translated into Norwegian and validated.^32^ In our study, missing items in the HADS were replaced based on recommended guidelines^33^ with valid scores for both subscales defined as at least five completed items. The sum score for participants who completed five or six items was calculated by multiplying the sum of the completed items by 7/5 or 7/6, respectively.

***General Self-Efficacy Scale (GSE)***

The GSE measures optimistic self-belief in coping with general life needs, tasks, and challenges. It consists of 10 statements rated by patients on a 4-point scale from 1 “completely agree” to 4 “completely disagree.” The total score is calculated by summing the individual item scores (range: 10–40), with higher scores indicating stronger self-efficacy.^34^ The GSE has demonstrated high reliability and validity^35,36^ and the Norwegian translation has been validated.^37,38^

***Fatigue questionnaire (FQ)***

The FQ was developed by Chalder et al.,^39^ and measures the extent and severity of fatigue. It consists of 11 items, seven physical and four mental. Each score was dichotomized and summarized, with a score > 4 defining substantial fatigue (SF). The questionnaire has been translated into Norwegian and validated^40^ for various groups, including Norwegian patients with IBD.^41^

**Statistical analyses**

Continuous data are presented as medians and ranges for variables with skewed distribution and means and standard deviations for normally distributed data. Categorical variables are presented as numbers and percentages. Crude between-group comparisons were performed using independent t-tests for normally distributed data or Mann–Whitney U tests for variables with skewed distributions. Categorical variables were compared using the chi-squared or Fisher’s exact test, as appropriate. The proportion of anxiety and depression was assessed using the HADS, with selected analyses stratified by gender. A cut‑off score of ≥8 on each subscale was used to indicate the presence of possible anxiety or depressive symptoms.^31,42^ We note that HADS is a screening instrument rather than a diagnostic tool; a lower cut‑off (≥8) prioritizes sensitivity for case‑finding, whereas a higher cut‑off (≥11) identifies probable cases with greater specificity. A descriptive table of HADS scores in CD and UC with cut‑off of ≥8-10 and ≥11 is provided in the supplementary data. Point estimates are provided with 95% confidence intervals (CIs), derived using binomial approximation.^43^

Robust multiple linear regression analyses were executed to compare HADS scores in patients with CD and UC in IBSEN III with those in the Norwegian general population in HUNT4, and adjusted for age and education. All analyses were performed separately for patients with UC and CD and stratified by gender. Results are reported as regression coefficients B with 95% CIs.

Logistic regression analyses were performed to explore the associations between anxiety and depression (dependent variables) and select possible predictive factors. Socio-demographic variables, SF, GSE, and clinical variables with a statistical significance of p ≤ 0.1 in univariate regression analyses were included in the multiple models. Collinearity between potential explanatory variables was assessed. The final multiple model was derived using backward variable selection starting with a full model that included all variables that reached p<0.10 in univariate analyses. Effect estimates are presented as odds ratios (OR) with 95% CI. Statistical significance level was set at p < 0.05 for the multiple regression analyses. All analyses were considered exploratory; therefore, no corrections were applied for multiple testing. Data were analyzed using IBM SPSS version 28 (IBM Corp., Armonk, NY, USA) and Stata version 18 (StataCorp. College Station, TX, USA), figure 2 was made in R version 4.5.0 (R Core Team, Vienna Austria).

**Ethical considerations**

This study was approved by the Regional Committee for Medical and Health Research Ethics in Southeast Norway (reference number: 2015/946). The HUNT Research Center at the Norwegian University of Science and Technology obtained permission from the Norwegian Data Inspectorate to store and handle data. Informed consent was obtained from all study participants prior to inclusion in both HUNT and IBSEN III. The IBSEN III study was registered at clinicaltrials.gov (NCT02727959).

**RESULTS**

**Study populations**

In total, 1562 adult (>18 years) patients with IBD were included in IBSEN III (UC: n=1003; CD: n=506) (Fig. 1). Of these, 938 (62.1%) completed the HADS at baseline. Compared with non-responders (n=564) or those with incomplete HADS data (n=7), HADS responders included a significantly higher proportion of females (51.3% vs. 43.7%, p=0.004) and a smaller proportion of current smokers (6.8% vs. 12.6%, p=0.004).

Table 1 shows the socio-demographic and clinical characteristics of the IBSEN III and HUNT4 survey cohort. The distribution of age, gender, and educational level in the patient population differed significantly from that in the general population (n=41916). Patients with IBD were significantly younger and had a higher level of education, and there was a higher proportion of males among patients with UC.

**Proportion of symptoms of anxiety and depression in patients with CD and UC compared with the general population (NGP)**

In the total sample, the proportion of anxiety was 37.4% [95% CI 32.0‒43.0%] in patients with CD and 32.1% [95% CI 28.0‒35.0%] in patients with UC, whereas depression was reported in 21.9% [95% CI 17.0‒27.0] and 16.8% [95%CI 14.0‒20.0%] in CD and UC, respectively. No statistically significant differences in anxiety and depression levels were observed between patients with UC and CD, however, both diagnostic groups exhibited significantly higher levels of anxiety and depression than those in the Norwegian general population (Table 2). A descriptive table of HADS scores in CD and UC with cut‑off of ≥8-10 and ≥11 is provided in the supplementary data.

Figures 2a and 2b illustrate the proportion of anxiety and depression in the IBD patients compared with NGP, stratified by gender. Compared with males in the NGP, males with CD had a significantly higher proportion of both anxiety and depression symptoms, whereas males with UC had a significantly higher proportion of symptoms of anxiety, but not depression. Females with CD and UC exhibited a significantly higher proportion of both anxiety and depression symptoms than females in the general population. These gender differences remained statistically significant after adjusting for age and education in the robust multiple linear regression analysis (Table 3).

**Factors associated with anxiety and depression in CD**

The results of the univariate and multiple logistic regression analyses are presented in Table 4. When adjusted for age, gender, HBI, SF and GSE, the final multiple logistic regression models indicated that SF increased the odds of anxiety fourfold (OR=4.1 [95% CI 2.1-7.8]), whereas GSE scores reduced the odds by 10% (OR=0.9 [95%CI 0.9-0.9]). When adjusted for age, marital status, education, work status HBI, SF and GSE, the final multiple logistic regression model indicated that patients with SF had more than 12 times higher odds of depression (OR=12.3 [95%CI 3.5‒43.3]). Moreover, unemployed patients had nearly four times higher odds for depression (OR= 3.8 [95%CI: 1,7‒8,6]) compared to employed patients, whereas GSE scores reduced the odds by 10% (OR=0.9 [95% 0.8-1.0].

**Factors associated with anxiety and depression in UC**

The results of the univariate and multiple logistic regression analyses for patients with UC are presented in table 5. When adjusted for age, gender, disease extent, SF and GSE scores, the final multiple logistic regression models showed that female gender increased the odds of anxiety by 50% (OR=1.5 [95% CI 1.0-2.3]), and SF increased the odds of anxiety fourfold (OR: 4.5 95% [CI: 2.8‒7.1]). Disease extent (left-sided colitis) and GSE scores were associated with reduced odds of anxiety. When adjusted for gender, education, work status, SF and GSE scores, patients with SF had more than 10 times higher odds of depression (OR= 10.2 [95% CI: 4.6‒22.7]), whereas higher GSE scores significantly reduced the risk of depression by 10% (OR= 0.9 [95% CI: 0.8‒0.9]).

**DISCUSSION**

In this study among newly diagnosed patients with IBD, we observed a significantly higher proportion of symptoms of anxiety and depression compared with the general Norwegian population. These findings are consistent with a systematic review on elevated rates of anxiety and depression in patients with IBD relative to the general population.^14^ In general, The HUNT population is considered to be representative of the Norwegian general population,^44^ however the distribution of age, gender, and educational level in the present patient population differed significantly from that in the general population in HUNT4. Our analyses of anxiety and depression based on gender and diagnosis, as well as comparisons with the Norwegian general population, and the use of robust regression analyses adjusted for age and educational level, strengthen the validity of our findings. To our knowledge, studies comparing newly diagnosed patients with IBD and general population, are scarce. Our study revealed gender-related differences. Compared to the Norwegian general population newly diagnosed females with IBD had significantly higher proportion of both anxiety and depression, whereas males with IBD had a significantly higher proportion of anxiety symptoms, but not depression. Our findings may reflect the challenging situation of being diagnosed with a chronic disease for both genders and further research is needed to investigate whether these findings persist or change over time. Moreover, a bidirectional relationship between IBD, anxiety, and depression has been suggested, with evidence indicating an increased risk of depression and anxiety both before and after IBD diagnosis.^45^ No significant differences in the proportion of symptoms of anxiety and depression were observed between patients with CD and UC, but when analyzed by gender, women with IBD had a significantly higher proportion of anxiety symptoms, aligning with earlier reviews.^7,14,46^

The proportion of anxiety and depression align with a prospective multi-center cohort study of patients newly diagnosed with IBD (< 6 months), which also employed the HADS with a cut-off score of ≥ 8 to indicate symptoms of anxiety or depression.^20^ In addition, a small study of newly diagnosed patients with IBD reported lower rates than those observed in our study^21^, while another study focusing on patients with UC found a higher proportion of depression than that observed in this study.^47^ A recent study^23^ applied a more rigorous HADS cut-off score of ≥ 11 when assessing symptoms of anxiety and depression in newly diagnosed patients. We chose a HADS cut‑off of ≥8 to identify possible symptoms of anxiety and depression, consistent with prior IBD and population studies. This approach favors case‑finding^42^ and comparability but will yield higher proportion estimates than a more conservative HADS cut‑off (≥11), which is often used to indicate probable clinical cases. Using the more rigorous cut-off yielded lower proportions of anxiety and depression symptoms in our study compared to those reported in the comparative study.^23^

However, comparing the findings from these studies with selected samples to those of our population-based cohort is complicated by differences in sample size, disease severity, and HADS cut-off scores, and neither of these studies compared rates with general populations.

In our study, substantial fatigue and self-efficacy were the strongest predictive factors independently associated with symptoms of anxiety and depression. Fatigue is a common symptom of many chronic illnesses, including IBD with nearly two-thirds of patients newly diagnosed with IBD reporting substantial fatigue.^48^ Fatigue is considered to be one of the most troublesome symptoms experienced by patients with IBD^49,50^ and negatively affects HRQoL^51^, thereby also affecting the patients` psychological well-being. Fatigue is also associated with anxiety and depression in IBD.^48,52-54^ The pathogenesis of fatigue remains unclear. However, psychological morbidities have been suggested as contributing factors,^55^ and there is a considerable symptomatic overlap between fatigue, anxiety, and depression.^16,54,56^ Nevertheless, our findings highlight the importance of recognizing fatigue as a potential contributor to anxiety and depression symptoms in newly diagnosed patients, despite limited treatment options owing to its unclear and multifactorial pathogenesis.^55^ In our study, patients with CD and SF had 12 times higher odds for symptoms of depression, however, the wide CIs (95% CI: 3,5<43,3) indicate considerable uncertainties, probably related to the low number of patients with CD and depression.

General self-efficacy refers to an individual`s belief in their ability to handle challenges^57^ and is associated with vulnerability to psychological distress.^57,58^ In addition, self-efficacy has been associated with effective self-management and self-esteem^59,60^ and studies in other chronic conditions have demonstrated that higher self-efficacy scores are associated with improved health outcomes.^61-63^ Our results suggest that higher self-efficacy is associated with lower odds of anxiety and depression symptoms, consistent with findings from other studies in patients with IBD.^64,65^ According to Bandura, self-efficacy is a modifiable factor,^57^ suggesting that interventions aimed at increasing self-efficacy may be particularly relevant for patients newly diagnosed with chronic diseases, such as IBD.

Our multiple logistic regression analyses indicated that work status was significantly associated with symptoms of depression in patients with CD. Inability to participate in work life may affect psychological well-being, and this finding partially aligns with previous studies,^15,66^ and a recent systematic review and meta-analysis^67^ on work impairment found that patients with CD were more negatively affected compared with patients with UC.

Interestingly, even though approximately 50% had active disease at diagnosis in our study, we did not find an association between IBD disease activity and the proportion of depression and anxiety symptoms, in contrast to previous systematic reviews.^7,14^ Contrary to the newly diagnosed patients in our study, these reviews included patients with a wide range of disease durations, which makes direct comparison challenging. This finding emphasizes the need to focus on the patient’s symptoms, in addition to disease activity, in the follow-up of the newly diagnosed patients. Early detection of psychological distress, regardless of disease activity, provides an opportunity for personalized symptom management and support. This is in accordance with recommendations in Mikocka-Walus et al with an integrated biopsychosocial care model including regular mental health screenings.^68^

A major strength of this study is its large, population-based sample and the comparison of newly diagnosed patients with IBD with age- and gender-adjusted cohorts from the renown general Norwegian population of HUNT4, providing a novel and unique insight into this patient population. Contrary to our study, disease activity in IBD studies is often assessed using clinical disease activity indices derived from self-reported data rather than biomarkers.^7^ Therefore, the inclusion of fecal calprotectin as a biomarker in this study is a notable strength. This study also has its limitations. Recall bias associated with patient-reported outcome measures cannot be entirely excluded. However, the large number of patients included in the IBSEN III study, and the similarity between HADS responders and non-responders across most background variables, strengthen our findings and support the representativeness of the study population with IBD. Furthermore, no data on symptoms of anxiety and depression among the IBSEN III patients prior to inclusion were available, and finally, the limited sample size of patients with CD and depression may limit the generalization of these results to the general CD patient population. It is also important to emphasize that the HADS is a screening tool, not a diagnostic tool, and HADS scores ≥ 8 do not indicate that the criteria for a clinical diagnosis of anxiety or depression (e.g., ICD-10 or DSM-5) are met.

In conclusion, this study provides valuable insights into the psychological challenges faced by patients newly diagnosed with IBD, emphasizing the importance of comprehensive care that addresses both the physical and psychological aspects of this chronic disease. Recognizing the gender-specific aspects of IBD is important for improving disease management and promoting personalized care. Such interventions may include addressing substantial fatigue, enhancing self-efficacy, and providing psychosocial support. Future longitudinal studies should explore the relationships between disease activity, psychological treatment regimen and interventions, as well as psychological outcomes to further advance understanding and improve treatment strategies. The findings in this study emphasize the importance of integrating psychological health screening and support into the routine care of newly diagnosed patients with IBD. Healthcare providers should prioritize early identification of psychological health challenges to enable timely and targeted interventions.

AUTHORS CONTRIBUTIONS

Conception and design of the study: MCH, MLH, GHH, TED, AWM, RO, LPJJ, VK, STL and IJ. Data collection: TBA, ØA, MBB, RB, TED, SOF, KAH, ØH, GHH, MLH, IJ, VAK, CL, BCO, RO, VS, RT and SV. Statistical analysis: MCH, RO and IJ. Analysis and interpretation of data and draft of the manuscript: MCH, RO, LPJJ, STL, and IJ. All authors critically revised the final manuscript for intellectual content and approval for publication.

CONFLICTS OF INTEREST

IJ, MCH, STL, TAa, ØA, MBB, RB, KAH, ØH, GHH, CL, AWM, BCO, RT, SV, LPJJ and RO declares no conflicts of interest. TED: has served as a speaker, consultant or advisory board member for AbbVie, Ferring, Johnson&Johnson, Pfizer, Pharmacosmos, Takeda, Tillotts, and Vifor Pharma. He has received unrestricted research grants from AbbVie, and Pharmacosmos. SOF: Personal fees from Takeda, Galapagos, Jansen-Cilag, Abbvie, Pharmacosmos, and Bristol-Myers-Squibb. M.L.H: Investigator-initiated research grants from Takeda, Pfizer, Tilllotts, Ferring, and Janssen. Speaker honoraria from Takeda, Tillotts, Ferring, AbbVie, Galapagos, MSD, and Meda. Advisory board for Takeda, Galapagos, MSD, Lilly, Janssen, Pfizer, and AbbVie. VAK: Consultant for Janssen-Cilag, scientific advisory board, and consultant for Takeda, speaker for Thermo Fischer, advisory board for Tillotts Pharma. VS: Sponsored by funds from Takeda.

DATA AVAILABILITY STATEMENT

The data for this study are stored on a secure server for sensitive data managed by the University of Oslo, Norway. The data underlying this article cannot be shared publicly due to the privacy of individuals that participated in the study. The data will be shared at reasonable request to the corresponding author.

FUNDING

This work was supported by Østfold University College. The IBSEN -III was supported by an investigator-initiated research grant from Takeda Pharmaceuticals and non-restricted research grants from Pfizer, Ferring Pharmaceuticals, Tillots Pharma, Foundation Dam, and the South-Eastern Health Authorities in Norway. This study and the IBSEN-III are investigator-initiated, and the sponsors did not play a role in the design, data collection, analysis, data interpretation, writing of the article or publication.

ACKNOWLEDGEMENTS

The authors thank the patients participating in the study, and furthermore, study coordinator Elisabeth Finnes, local study nurses and investigators who contributed to inclusion and follow-up of patients in IBSEN-III. The Trøndelag Health study is a collaboration between the HUNT Research Center (Faculty of Medicine and Health Sciences, Norwegian University of Science and Technology, NTNU), Trøndelag County Council, Central Norway Regional Health Authority, and the Norwegian Institute of Public Health, and we gratefully acknowledge their contributions. We thank Unger-Vetlesen Institute for analyzing the feacel samples. We thank Sigurd Maurud for aid in designing figures 2a and 2b.

**References**

1. Baumgart DC, Carding SR. Inflammatory bowel disease: Cause and immunobiology. *The Lancet* 2007;**369**:1627-40.

2. Abraham C, Cho J. Inflammatory bowel disease. *N Engl J Med* 2009;**361**:2066-78.

3. Cohen RD. The quality of life in patients with crohn's disease. *Aliment Pharmacol Ther* 2002;**16**:1603-9.

4. Knowles SR, Keefer L, Wilding H*, et al.* Quality of life in inflammatory bowel disease: A systematic review and meta-analyses-part ii. *Inflamm Bowel Dis* 2018;**24**:966-76.

5. Slonim-Nevo V, Sarid O, Friger M*, et al.* Effect of social support on psychological distress and disease activity in inflammatory bowel disease patients. *Inflamm Bowel Dis* 2018;**24**:1389-400.

6. Knowles SR, Graff LA, Wilding H*, et al.* Quality of life in inflammatory bowel disease: A systematic review and meta-analyses—part i. *Inflamm Bowel Dis* 2018;**24**:742-51.

7. Barberio B, Zamani M, Black CJ, Savarino EV, Ford AC. Prevalence of symptoms of anxiety and depression in patients with inflammatory bowel disease: A systematic review and meta-analysis. *Lancet Gastroenterol Hepatol* 2021;**6**:359-70.

8. Eugenicos MP, Ferreira NB. Psychological factors associated with inflammatory bowel disease. *Br Med Bull* 2021;**138**:16-28.

9. Van Assche G, Dignass A, Reinisch W*, et al.* The second european evidence-based consensus on the diagnosis and management of crohn's disease: Special situations. *J Crohns Colitis* 2010;**4**:63-101.

10. Van Assche G, Dignass A, Bokemeyer B*, et al.* The second european evidence-based consensus on the diagnosis and management of ulcerative colitis part 3: Special situations. *J Crohns Colitis* 2013;**7**:1-33.

11. Engel K, Homsi M, Suzuki R*, et al.* Newly diagnosed patients with inflammatory bowel disease: The relationship between perceived psychological support, health-related quality of life, and disease activity. *Health Equity* 2021;**5**:42-8.

12. Schoefs E, Vermeire S, Ferrante M*, et al.* What are the unmet needs and most relevant treatment outcomes according to patients with inflammatory bowel disease? A qualitative patient preference study. *J Crohns Colitis* 2023;**17**:379-88.

13. Ludvigsson JF, Olén O, Larsson H*, et al.* Association between inflammatory bowel disease and psychiatric morbidity and suicide: A swedish nationwide population-based cohort study with sibling comparisons. *J Crohns Colitis* 2021;**15**:1824-36.

14. Mikocka-Walus AA, Knowles S, Keefer L, Graff L. Controversies revisited : A systematic review of the comorbidity of depression and anxiety with inflammatory bowel diseases. *Inflamm Bowel Dis* 2016;**22**:752-62.

15. Nahon S, Lahmek P, Durance C*, et al.* Risk factors of anxiety and depression in inflammatory bowel disease. *Inflamm Bowel Dis* 2012;**18**:2086-91.

16. Chavarría C, Casanova MJ, Chaparro M*, et al.* Prevalence and factors associated with fatigue in patients with inflammatory bowel disease: A multicentre study. *J Crohns Colitis* 2019;**13**:996-1002.

17. Huppertz-Hauss G, Høivik ML, Jelsness-Jørgensen LP*, et al.* Fatigue in a population-based cohort of patients with inflammatory bowel disease 20 years after diagnosis: The ibsen study. *Scand J Gastroenterol* 2017;**52**:351-8.

18. Siegel CA, Dubinsky MC. Editorial: How to interpret risks and prediction of complications in crohn's disease - can our patients interpret them? Authors' reply. *Aliment Pharmacol Ther* 2016;**43**:653-.

19. Dubinsky MC, Dotan I, Rubin DT*, et al.* Burden of comorbid anxiety and depression in patients with inflammatory bowel disease: A systematic literature review. *Expert Rev Gastroenterol Hepatol* 2021;**15**:985-97.

20. Bernabeu P, Belén-Galipienso O, van-der Hofstadt C*, et al.* Psychological burden and quality of life in newly diagnosed inflammatory bowel disease patients. *Front Psychol* 2024;**15**:1334308.

21. McCombie AM, Mulder RT, Gearry RB. Coping strategies and psychological outcomes of patients with inflammatory bowel disease in the first 6 months after diagnosis. *Inflamm Bowel Dis* 2015;**21**:2272-80.

22. Kurina LM, Goldacre MJ, Yeates D, Gill LE. Depression and anxiety in people with inflammatory bowel disease. *J Epidemiol Community Health* 2001;**55**:716-20.

23. Riggott C, Gracie DJ, Ford AC. Prevalence and predictors of symptoms of anxiety or depression at diagnosis in patients with inflammatory bowel disease: An inception cohort. *Aliment Pharmacol Ther* 2025;**62**:788-98.

24. Kristensen VA, Opheim R, Perminow G*, et al.* Inflammatory bowel disease in south-eastern norway iii (ibsen iii): A new population-based inception cohort study from south-eastern norway. *Scand J Gastroenterol* 2021;**56**:899-905.

25. Kristensen V, Røseth A, Ahmad T, Skar V, Moum B. Fecal calprotectin: A reliable predictor of mucosal healing after treatment for active ulcerative colitis. *Gastroenterol Res Pract* 2017;**2017**:2098293.

26. Harvey RF, Bradshaw JM. A simple index of crohn`s-disease activity. *Lancet* 1980;**315**:514.

27. Walmsley RS, Ayres RCS, Pounder RE, Allan RN. A simple clinical colitis activity index. *Gut* 1998;**43**:29-32.

28. Peyrin-Biroulet L, Panés J, Sandborn WJ*, et al.* Defining disease severity in inflammatory bowel diseases: Current and future directions. *Clin Gastroenterol Hepatol* 2016;**14**:348-54.e17.

29. Satsangi J, Silverberg MS, Vermeire S, Colombel JF. The montreal classification of inflammatory bowel disease: Controversies, consensus, and implications. *Gut* 2006;**55**:749-53.

30. Åsvold BO, Langhammer A, Rehn TA*, et al.* Cohort profile update: The hunt study, norway. *Int J Epidemiol* 2023;**52**:e80-e91.

31. Zigmond AS, Snaith RP. The hospital anxiety and depression scale. *Acta Psychiatri Scand* 1983;**67**:361-70.

32. Mykletun A, Stordal E, Dahl AA. Hospital anxiety and depression (had) scale: Factor structure, item analyses and internal consistency in a large population. *Br J Psychiatry* 2001;**179**:540-4.

33. Bell ML, Fairclough DL, Fiero MH, Butow PN. Handling missing items in the hospital anxiety and depression scale (hads): A simulation study. *BMC Res Notes* 2016;**9**:479.

34. Schwarzer R, Jerusalem M. Generalized self-efficacy scale. . In: J. Weinman SW, & M. Johnston, editor. *Measures in health psychology: A user’s portfolio causal and control beliefs* Windsor, UK: NFER-NELSON.; 1995: 35-7.

35. Leganger A, Kraft P, Roysamb E. Perceived self-efficacy in health behaviour research: Conceptualisation, measurement and correlates. *Psychol Health* 2000;**15**:51-69.

36. Bonsaksen T, Lerdal A, Heir T*, et al.* General self-efficacy in the norwegian population: Differences and similarities between sociodemographic groups. *Scand J Public Health* 2019;**47**:695-704.

37. Røysamb E. *Adolescent risk making: Behaviour patterns and the role of emotions and cognitions*: University of Oslo; 1997.

38. Røysamb E, Schwarzer R, Jerusalem M. *Norwegian version of the general percieved self-efficacy scale* <http://userpage.fu-berlin.de/~health/norway.htm>, 1998.

39. Chalder T, Berelowitz G, Pawlikowska T*, et al.* Development of a fatigue scale. *J Psychosom Res* 1993;**37**:147-53.

40. Loge JH, Ekeberg Ø, Kaasa S. Fatigue in the general norwegian population: Normative data and associations. *J Psychosom Res* 1998;**45**:53-65.

41. Jelness-Jørgensen L-P, Bernklev T, Henriksen M, Torp R, Moum B. Chronic fatigue is more prevalent in patients with inflammatory bowel disease than in healthy controls *Inflammatory Bowel Diseases* 2011;**17**:1564-72.

42. Bjelland I, Dahl AA, Haug TT, Neckelmann D. The validity of the hospital anxiety and depression scale: An updated literature review. *Journal of Psychosomatic Research* 2002;**52**:69-77.

43. Brown LD, Cai TT, DasGupta A. Interval estimation for a binomial proportion. *Stat Sci* 2001;**16**:101-33.

44. Krokstad S, Langhammer A, Hveem K*, et al.* Cohort profile: The hunt study, norway. *Int J Epidemiol* 2013;**42**:968-77.

45. Bisgaard TH, Allin KH, Keefer L, Ananthakrishnan AN, Jess T. Depression and anxiety in inflammatory bowel disease: Epidemiology, mechanisms and treatment. *Nat Rev Gastroenterol Hepatol* 2022;**19**:717-26.

46. Graff LA, Geist R, Kuenzig ME*, et al.* The 2023 impact of inflammatory bowel disease in canada: Mental health and inflammatory bowel disease. *Journal of the Canadian Association of Gastroenterology* 2023;**6**:S64-S75.

47. Moon JR, Lee CK, Hong SN*, et al.* Unmet psychosocial needs of patients with newly diagnosed ulcerative colitis: Results from the nationwide prospective cohort study in korea. *Gut and Liver* 2020;**14**:459-67.

48. Holten KA, Bernklev T, Opheim R*, et al.* Fatigue in patients with newly diagnosed inflammatory bowel disease: Results from a prospective inception cohort, the ibsen iii study. *J Crohns Colitis* 2023;**17**:1781-90.

49. van Langenberg DR, Gibson PR. Systematic review: Fatigue in inflammatory bowel disease. *Aliment Pharmacol Ther* 2010;**32**:131-43.

50. Casellas F, López-Vivancos J, Vergara M, Malagelada J. Impact of inflammatory bowel disease on health-related quality of life. *Dig Dis* 1999;**17**:208-18.

51. Minderhoud IM, Oldenburg B, van Dam PS, van Berge Henegouwen GP. High prevalence of fatigue in quiescent inflammatory bowel disease is not related to adrenocortical insufficiency. *Am J Gastroenterol* 2003;**98**:1088-93.

52. Häuser W, Stallmach A, Kocalevent R-D, Rose M, Fliege H. Biopsychosocial predictors of fatigue in quiescent and mild ulcerative colitis-an explorative study. *Psychosoc Med* 2005;**2:Doc7**.

53. Stroie T, Preda C, Istratescu D*, et al.* Anxiety and depression in patients with inactive inflammatory bowel disease: The role of fatigue and health-related quality of life. *Medicine (Baltimore)* 2023;**102**:e33713.

54. Czuber-Dochan W, Ream E, Norton C. Review article: Description and management of fatigue in inflammatory bowel disease. *Aliment Pharmacol Ther* 2013;**37**:505-16.

55. Borren NZ, van der Woude CJ, Ananthakrishnan AN. Fatigue in ibd: Epidemiology, pathophysiology and management. *Nat Rev Gastroenterol Hepatol* 2019;**16**:247-59.

56. Romberg-Camps MJ, Bol Y, Dagnelie PC*, et al.* Fatigue and health-related quality of life in inflammatory bowel disease: Results from a population-based study in the netherlands: The ibd-south limburg cohort. *Inflamm Bowel Dis* 2010;**16**:2137-47.

57. Bandura A. Self-efficacy: Toward a unifying theory of behavioral change. *Psychol Rev* 1977;**84**:191-215.

58. Bandura A, Freeman WH, Lightsey R. Self-efficacy: The exercise of control. Springer, 1999.

59. Opheim R, Moum B, Grimstad BT*, et al.* Self-esteem in patients with inflammatory bowel disease. *Qual Life Res* 2020;**29**:1839-46.

60. Rosenberg M. *Concieving the self*. New York NY: Basic Books; 1979.

61. D'Souza MS, Karkada SN, Parahoo K*, et al.* Self-efficacy and self-care behaviours among adults with type 2 diabetes. *Appl Nurs Res* 2017;**36**:25-32.

62. Chen AMH, Yehle KS, Plake KS*, et al.* The role of health literacy, depression, disease knowledge, and self-efficacy in self-care among adults with heart failure: An updated model. *Heart Lung* 2020;**49**:702-8.

63. Selzler A-M, Moore V, Habash R*, et al.* The relationship between self-efficacy, functional exercise capacity and physical activity in people with copd: A systematic review and meta-analyses. *COPD: J Chronic Obstr Pulm Dis* 2020;**17**:452-61.

64. Izaguirre MR, Taft T, Keefer L. Validation of a self-efficacy scale for adolescents and young adults with inflammatory bowel disease. *J Pediatr Gastroenterol Nutr* 2017;**65**:546-50.

65. Eindor-Abarbanel A, Naftali T, Ruhimovich N*, et al.* Important relation between self-efficacy, sense of coherence, illness perceptions, depression and anxiety in patients with inflammatory bowel disease. *Frontline Gastroenterol* 2021;**12**:601-7.

66. De Boer AGEM, Bennebroek Evertsz’ F, Stokkers PC*, et al.* Employment status, difficulties at work and quality of life in inflammatory bowel disease patients. *Eur J Gastroenterol Hepatol* 2016;**28**:1130-6.

67. Youssef M, Hossein-Javaheri N, Hoxha T, Mallouk C, Tandon P. Work productivity impairment in persons with inflammatory bowel diseases: A systematic review and meta-analysis. *J Crohns Colitis* 2024;**18**:1486-504.

68. Mikocka-Walus A, Massuger W, Knowles SR*, et al.* Psychological distress is highly prevalent in inflammatory bowel disease: A survey of psychological needs and attitudes. *JGH Open* 2020;**4**:166-71.

**Figure captions**

Fig. 1: Patient enrolment flowchart. Abbreviations: IBD, Inflammatory bowel disease; CD, Crohn’s disease; UC, ulcerative colitis; HADS, Hospital Anxiety and Depression Scale.

Fig 2a: Proportion of anxiety (HADS-A ≥ 8 with 95% CI)

Males: Norwegian general population 13.1% **(**12.6-13-6), CD 27.3% (19.6-36.1), UC 22.0% (17.7-26.8)

Females: Norwegian general population 20.7 % (20.2-21.3), CD 44.3% (36.8-52.0), UC 41.3 (35.7-47.1)

Fig 2b: Proportion of depression (HADS-D ≥ 8 with 95% CI)

Males: Norwegian general population 10.2% (9.8-10.7), CD 18.2% (11.8-26.2), UC 13.7% (10.2-17.8)

Females: Norwegian general population 8.8% (8.5-9.2), CD 24.4 % (18.3-31.5), UC 20.3% (16.0-25.3)
