# Supplementary material for: Anxiety and depression in newly diagnosed patients with inflammatory bowel disease (the IBSEN III study) compared with the general population in Norway
Source: J Crohns Colitis. 2026 Mar 4;20(3):jjag021. doi: 10.1093/ecco-jcc/jjag021 (PMC13016783; doi:10.1093/ecco-jcc/jjag021)
Supplement: jjag021_Supplementary_Data [file jjag021_supplementary_data.zip › XXXSUPPLI-1.DOC]

| **Supplementary table 1:**  **Proportion of anxiety and depression in patients with Crohn’s disease and ulcerative colitis** | | | | |
| --- | --- | --- | --- | --- |
|  | **HADS-A 8-10** | **HADS-A ≥ 11** | **HADS-D 8-10** | **HADS- D ≥ 11** |
|  | n (%) | n (%) | n (%) | n (%) |
| **Crohn`s disease** (n= 297) | 55 (18.5) | 56 (18.9) | 41 (13.8) | 24 (8.1%) |
| **Ulcerative colitis** (n= 641) | 97 (15.1) | 103 (16.1) | 68 (10.6) | 40 (6.2) |
